# Optimal Design for a Novel Compliant XY Platform Integrated with a Hybrid Double Symmetric Amplifier Comprising One-Lever and Scott–Russell Mechanisms Arranged in a Perpendicular Series Layout for Vibration-Assisted CNC Milling

**DOI:** 10.3390/mi16070793

**Published:** 2025-07-03

**Authors:** Minh Phung Dang, Anh Kiet Luong, Hieu Giang Le, Chi Thien Tran

**Affiliations:** Faculty of Mechanical Engineering, Ho Chi Minh City University of Technology and Education, Ho Chi Minh City 700000, Vietnam

**Keywords:** compliant mechanism, Flexure 02-DOF mechanism, firefly algorithm, vibration-aided fine milling, surface morphology

## Abstract

Compliant mechanisms are often utilized in precise positioning systems but have not been thoroughly examined in vibration-aided fine CNC machining. This study aims to develop a new 02-DOF flexure stage for vibration-aided fine CNC milling. A hybrid displacement amplifier, featuring a two-lever mechanism, two Scott–Russell mechanisms, and a parallel leading mechanism, was integrated into a symmetric perpendicular series configuration to create an innovative design. The pseudo-rigid body model (PRBM), Lagrangian approach, finite element analysis (FEA), and Firefly optimization algorithm were employed to develop, verify, and optimize the quality response of the new positioner. The PRBM and Lagrangian methods were used to construct an analytical model, while finite element analysis was used to validate the theoretical solution. The primary natural frequency results from theoretical and FEM methods were 318.16 Hz and 308.79 Hz, respectively. The difference between these techniques was 3.04%, demonstrating a reliable modelling strategy. The Firefly optimization approach applied mathematical equations to enhance the key design factors of the mechanism. The prototype was then built, revealing an error of 7.23% between the experimental and simulated frequencies of 331.116 Hz and 308.79 Hz, respectively. The specimen was subsequently mounted on the fabricated optimization positioner, and vibration-assisted fine CNC milling was performed at 100–1000 Hz. At 400 Hz, the specimen achieved ideal surface roughness with a Ra value of 0.187 µm. The developed design is a potential structure that generates non-resonant frequency power for vibration-aided fine CNC milling.

## 1. Introduction

Currently, compliant mechanisms have evolved swiftly and are recognized for their vast utility across several fields, including positioning systems [1,2,3], soft grippers [4,5,6,7], bistable mechanisms [8], a constant-torque mechanism [9], and vibration-assisted machining [10,11,12]. In particular, within the vibration-assisted machining (VAM) field, Gu et al. [13] developed a new compliant XY positioner integrated with a symmetric one-lever displacement amplifier, featuring a working stroke of 38 µm × 42 µm for vibration-assisted roller polishing on silicon carbide. Gu et al. [14] proposed a novel flexure 02-DOF positioner combined with a symmetric two-lever displacement amplifier with a working travel of 70 μm × 75 μm. Cheng et al. [15] developed a new compliant positioner integrated with a hybrid amplifier using a bridge-lever mechanism for vibration-assisted polishing. In this research, the working stroke of the proposed positioner was 71 μm × 83 μm, and the X–Y direction natural frequencies were 205.39 Hz and 237.51 Hz. Hung et al. [16] developed a new compliant positioner with a symmetric three-lever displacement amplifier for vibration-assisted polishing. Qin et al. [17] proposed a compliant end-effector structure based on flexure beams for robotic polishing. The proposed structure’s equivalent stiffness and resonant frequency were 0.335 N/μm and 42.1 Hz, respectively. Zhang et al. [18] developed a compliant passive constant-force mechanism for optical reflecting mirror robotic polishing.

In the vibration-assisted milling field [19], Tuan et al. [20] developed a compliant XY positioner integrated with an asymmetric one-lever displacement amplifier for non-resonant vibration-assisted milling of the AL-6061 specimen. In this research, the authors employed a hybrid approach combining Six Sigma and the nondominated sorting genetic algorithm to optimize the main parameters of the proposed structure. The optimized FEA resonant frequency was 641.2 Hz. The investigated frequencies ranged from 0 to 700 Hz, with the optimal experimental frequency being 325 Hz and a surface roughness of 1.13 µm. Dang et al. [21] created a novel flexure 02-DOF structure integrated with a symmetric four-lever displacement amplifier for non-resonant vibration-aided fine CNC milling of the 2083 hard specimen. The optimized first natural frequency based on FEA analysis was 1216.6 Hz. The examined frequencies ranged from 700 to 1000 Hz, and the optimal experimental frequency of the developed mechanism was 900 Hz with a surface roughness of 0.321 µm.

Concerning the studied references on compliant XY positioners, numerous researchers have developed various types of structural designs based on PEA actuators [22]. Meanwhile, the working stroke of the PEA actuators is limited. Therefore, the compliant displacement amplifiers [23], including the symmetric bridge-lever mechanism [24], a symmetric double rocker mechanism [25], a symmetric two-lever mechanism [26], a symmetric three-lever mechanism [27], a bridge mechanism [28,29], and a Scott–Russell mechanism [30], were developed to enhance the working stroke of the XY positioners. Meanwhile, a hybrid double symmetric amplifier comprising one lever and Scott–Russell mechanisms arranged in a perpendicular series layout has not been investigated for a flexure 02-DOF mechanism applied for vibration-aided milling.

There are three configurations: a series layout, a parallel layout, and a series–parallel layout for building structural designs for compliant XY positioners [22]. Meanwhile, a series–parallel configuration is suitable for obtaining high-quality responses to develop compliant XY positioners based on PEA actuators. Wu et al. [31] developed a new XY positioner based on the series–parallel configuration with an operational travel of 28.27 µm × 27.62 µm. To date, there has been a rapid advancement in 02-DOF compliant mechanisms. Conversely, there is a significant gap in terms of comprehensive studies aiming to develop vibration-assisted CNC milling procedures, including enhancing the high-output working stroke, fabricated specimen surface quality and suitable resonant frequency of the developed flexure positioner. Therefore, this study presents a novel compliant XY positioner integrated with a hybrid displacement amplifier consisting of four-lever and Scott–Russell mechanisms, based on a symmetric perpendicular series layout for potential application in a non-resonant vibration-assisted milling process.

Additionally, a significant issue concerns modelling the behaviors of 02-DOF compliant mechanisms. Various analytical approaches [32,33] now exist for analyzing the operational characteristics of flexure XY positioners. These practical analytical methods comprise the compliance matrix method [14,34], the beam constraint model [35,36], and the pseudo-rigid-body model (PRBM) [37,38]. Among these analytical approaches, the hybrid method combining the PRBM with the Lagrange technique is an efficient methodology for modelling the static–dynamic characteristics of the compliant XY positioner. In addition, some studies are based on the intelligent behaviors and high convergence speed of the Firefly algorithm [39]. Therefore, this study applied the hybrid conceptual framework of PRBM, the Lagrange technique, FEA, and the Firefly algorithm for the modelling, validation, and optimization of the main parameters of the developed positioner.

This research aims to develop a novel compliant XY structure that integrates a hybrid double symmetric amplifier utilizing one-lever and Scott–Russell mechanisms, arranged in an alternating perpendicular series layout, with potential applications in non-resonant vibration-assisted fine CNC milling. The static–dynamic features of the new design were determined using the PRBM and Lagrange methods. The effectiveness of the developed positioner was enhanced utilizing the Firefly algorithm. The prototype of the optimal design was fabricated using the wire electrical discharge machining method. A physical assessment confirmed the correctness of the analytical and FEA findings. The experimental vibration-assisted CNC milling method was executed based on the actual prototype for the SKD11 specimen. This research used an experimental method to evaluate surface roughness and surface morphology, as well as determining the appropriate frequency for the constructed positioner.

## 2. Mechanical Design

**Figure 1:**
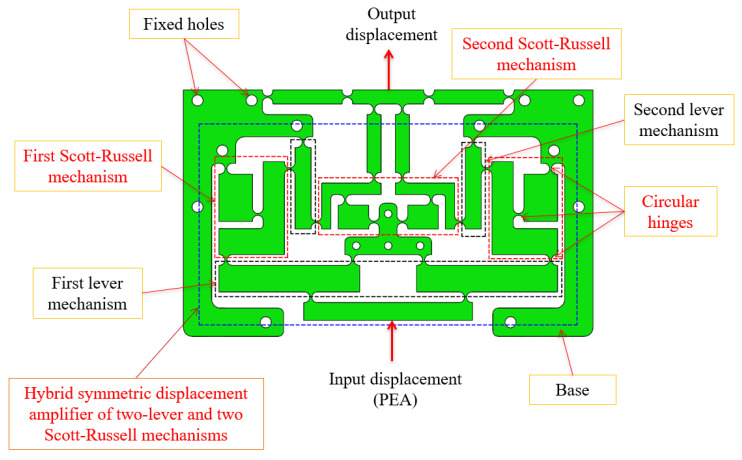
Hybrid symmetric amplifier of two-lever and two Scott–Russell mechanisms.

This research aims to create a novel vibration-assisted compliant positioner for use in CNC milling that enhances the surface roughness of finely produced items. Furthermore, using the designed compliant positioner, a non-resonant approach generates vibrations within the specimen. Therefore, the symmetric structures were proposed for developing the design of X and Y clusters of the compliant positioner to ensure the controlled position in the vibration-assisted CNC milling process. In addition, the specimen was mounted on the compliant positioner to conduct the vibration-assisted CNC milling process to evaluate the effectiveness of the developed positioner. As shown in Figure 1, the structure is explicitly constructed using a hybrid double symmetric amplifier, which consists of a two-lever mechanism and two Scott–Russell mechanisms arranged in a perpendicular series layout, integrated with right circular hinges to achieve maximum rotational center accuracy and minimize parasitic motion error. The integration of a symmetric displacement amplifier, featuring four floors arranged in an alternating perpendicular series architecture, is depicted in Figure 1.

**Figure 2:**
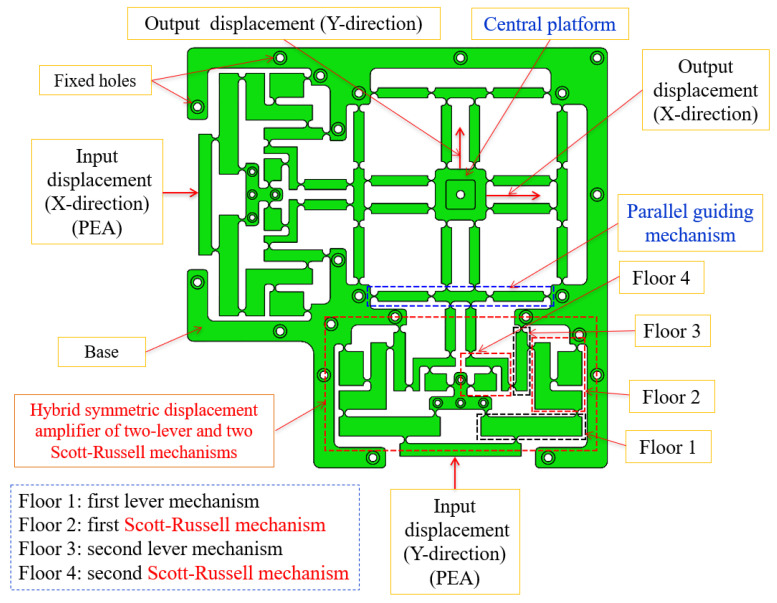
Developed flexure XY positioning structure.

**Figure 3:**
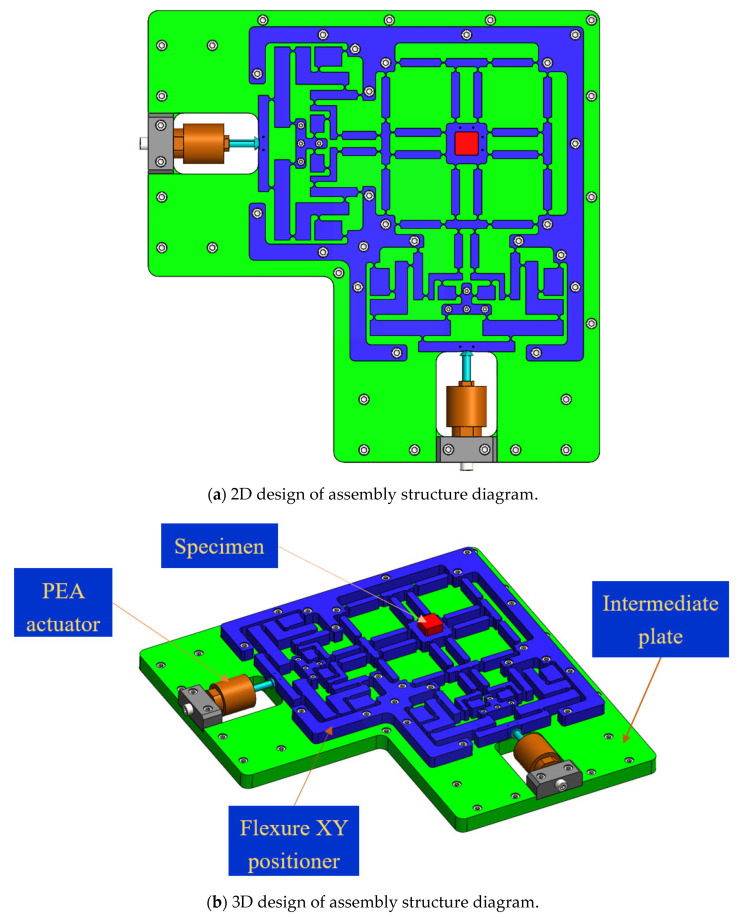
2D and 3D assembly design diagrams of the assembly cluster.

**Figure 4:**
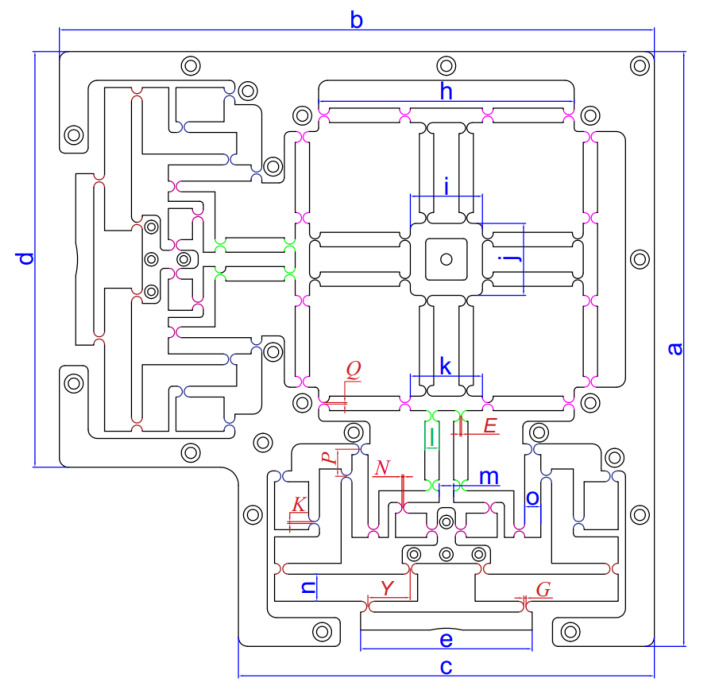
A schema demonstrating the initial design parameters of the suggested structure.

The developed XY platform can generate vibrations independently or concurrently by employing two PEA actuators oriented perpendicularly, as exhibited in Figure 2. The 02-DOF positioner features a hybrid double symmetric amplifier comprising one lever and Scott–Russell mechanisms arranged in a perpendicular series layout, an equivalent leading structure, and an inner stand, as illustrated in Figure 2. The equivalent leading structure utilizes circular flexure joints to minimize decoupling mobility imprecision and enhance the stiffness of the proposed positioner. The 2D-3D assembly design diagrams of the developed positioner are shown in Figure 3a,b. Figure 4 displays the primary dimensions of the platform. As presented in Table 1, the principal dimensional values of the developed positioner are measured in millimetres. The overall sizes of the proposed mechanism are 330.5 × 330.5 × 15 mm.

## 3. Proposed Hybrid Method

**Figure 5:**
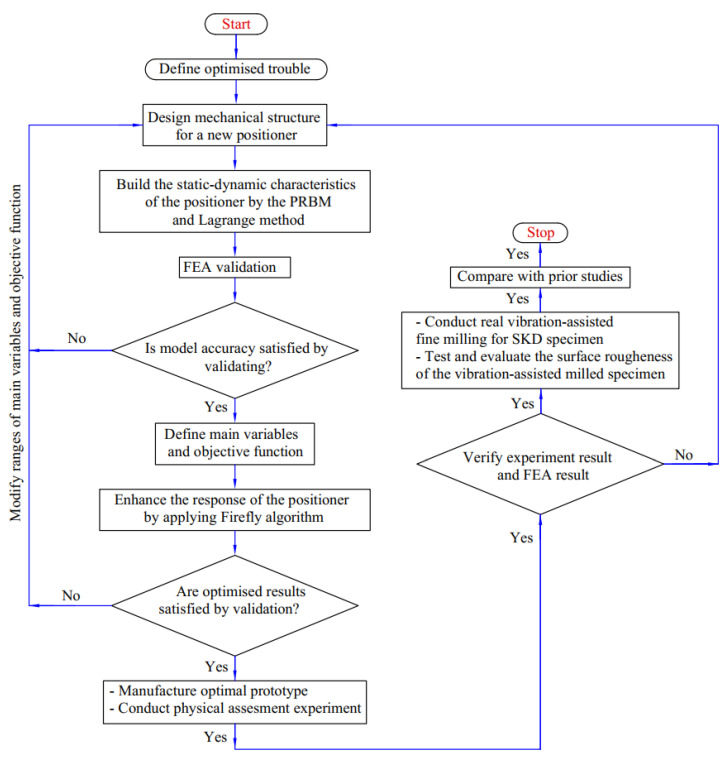
A schematic depicting the suggested optimization combination approach for the developed mechanism.

The novel compliant positioner was developed using a hybrid double symmetric amplifier that combines one-lever and Scott–Russell mechanisms, arranged in an alternating perpendicular series layout for vibration-assisted CNC milling. The static–dynamic features of the flexure positioner were modelled using the PRBM and the Lagrangian methods. Next, the Firefly algorithm [36] was applied to enhance the proposed positioner’s response quality. Figure 5 depicts the flowchart of the offered hybrid conceptual optimization framework strategy for the developed positioner. More details about the specific steps of the hybrid optimization approach are provided below.

(i) For the SKD11 non-resonant vibration-assisted fine milling, we developed a novel compliant positioner integrated with a new hybrid displacement amplifier with good technical performance.
(ii) The static–dynamic characteristics of the proposed positioner were built using the integration method of the PRBM and Lagrange technique.
(iii) The analytical results of the quality response were verified using FEA analysis.
(iv) The main parameters, the objective function, and limitations for design variables and the objective function were defined.
(v) We optimized the proposed positioner’s main parameters to enhance the developed positioner’s quality response using the Firefly algorithm.
(vi) The optimized analytical results were validated via FEA analysis.
(vii) We fabricated the optimal prototype based on optimal design variables.
(viii) A physical assessment experiment was conducted to verify the optimal results.
(ix) We conducted real non-resonant vibration-assisted fine milling for the SKD 11 specimen in a frequency range [100 Hz, 1000 Hz].
(x) We tested and evaluated the surface roughness of the vibration-assisted milled specimens.
(xi) Comparisons with prior studies were undertaken.

## 4. Results Analysis and Discussion

### 4.1. Analytical Model Investigation

**Figure 6:**
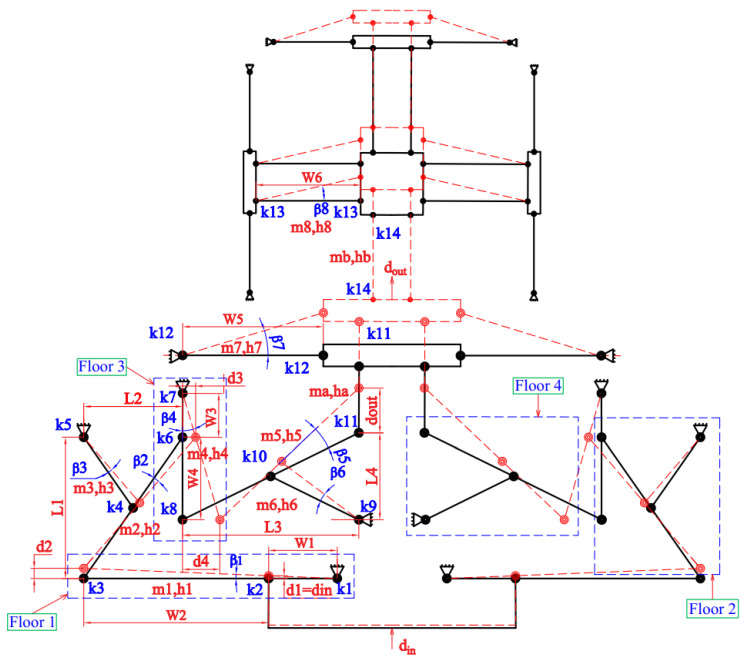
A PRBM diagram for modelling the proposed compliant positioner.

First, the static–dynamic characteristics of the proposed positioner were established using the PRBM and Lagrange techniques. Next, the analytical results were verified via the FEA analysis. The PRBM diagram for the proposed positioner is illustrated in Figure 6.

**Figure 7:**
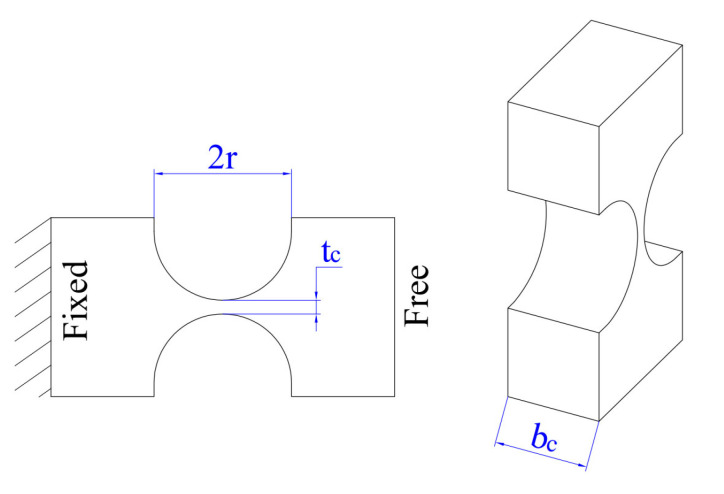
Main parameters of the right circular hinge.

Because of their high center accuracy, the right circular joints were suggested for integration into the created positioner to enhance the stage’s stiffness and resonant frequency, as well as eliminating the parasitic motion error for high positioning precision. Figure 7 demonstrates that the critical parameters of the employed right circular hinge are thickness ($t_{c}$), radius (*r*), and width ($b_{c}$).

As demonstrated in Figure 2 and Figure 6, $d_{in}$ and $d_{out}$ were the input–output displacement values of the displacement amplifier. The movement energy transfer diagram of the hybrid displacement amplifier is illustrated in Figure 6. More specifically, the first floor’s output displacement was the second floor’s input displacement. Similarly, the second floor’s output movement was the third floor’s input, and the third floor’s output movement was the fourth floor’s input. Finally, the fourth floor’s output movement was the hybrid amplifier’s output movement. As illustrated in Figure 6, the output displacements of four floors and the amplification ratio were calculated using the following Equations:

(1) $$d_{1}=d_{in}$$

(2) $$d_{2}=\frac{(W_{1}+W_{2})d_{in}}{W_{1}}$$

(3) $$d_{3}=\frac{d_{2}L_{1}}{L_{2}}=\frac{(W_{1}+W_{2})L_{1}d_{in}}{W_{1}L_{2}}$$

(4) $$d_{4}=\frac{d_{3}(W_{3}+W_{4})}{H_{3}}=\frac{(W_{1}+W_{2})(W_{3}+W_{4})L_{1}d_{in}}{W_{1}W_{3}L_{2}}$$

(5) $$d_{out}=\frac{d_{4}L_{3}}{L_{4}}=\frac{L_{3}(W_{1}+W_{2})(W_{3}+W_{4})L_{1}d_{in}}{L_{4}H_{1}H_{3}L_{2}}$$

(6) $$AR=\frac{d_{out}}{d_{in}}=\frac{(W_{1}+W_{2})(W_{3}+W_{4})L_{1}L_{3}}{W_{1}W_{3}L_{2}L_{4}}$$

where *W*_i_ (i = 1, 2, …, 5, 6) and *L*_q_ (*q* = 1, 2, 3, 4) indicate the distances in the horizontal direction and vertical direction, respectively; $\beta_{j}$ (*j* = 1, 2,…, 7, 8) denotes the rigid-link rotary angles, and *m*_i_ denotes the mass. In addition, for right circular hinges, the torsion stiffness ($K_{i}$) and inertia moment ($I_{i}$) of the rigid links are calculated using the following Equations (7) and (8), respectively:

(7) $$K_{c}=\frac{2Eb_{c}t_{c}^{2.5}}{9\pi r^{0.5}}$$

(8) $$I_{j}=\frac{m_{i}W_{j}^{2}}{12}$$

where the main parameters of the right circular hinge, $t_{i}$ and $r_{i}$, denote thickness and radius, and E and $b_{i}$ denote the Young modulus and width, respectively.

The kinetic energy of the recommended mechanism was calculated according to Equation (9):

(9) $$E_{k}=\sum_{1}^{n}(E_{t}+E_{r})=\sum_{1}^{8}(E_{t}+E_{r})=\sum_{1}^{8}(\frac{1}{2}m_{i}v_{i}^{2}+\frac{1}{2}I_{i}{\overset{\cdot }{\beta }}_{j}^{2})$$

The equation for determining the kinetic energy of every stiff connection is expressed as follows:

(10) $$\begin{array}{l} E_{k}=\left[\frac{1}{2}m_{1}{\left(\frac{W_{1}+W_{2}}{2}\right)}^{2}+\frac{1}{2}I_{1}\right]{\dot{\beta }}_{1}^{2}+\left[\frac{1}{2}m_{2}{\left(L_{BC}\right)}^{2}+\frac{1}{2}I_{2}\right]{\dot{\beta }}_{2}^{2}\\ +\left[\frac{1}{2}m_{3}{\left(L_{CD}\right)}^{2}+\frac{1}{2}I_{3}\right]{\dot{\beta }}_{3}^{2}+\left[\frac{1}{2}m_{4}{\left(\frac{W_{3}+W_{4}}{2}\right)}^{2}+\frac{1}{2}I_{4}\right]{\dot{\beta }}_{4}^{2}\\ +\left[\frac{1}{2}m_{5}{\left(L_{FG}\right)}^{2}+\frac{1}{2}I_{5}\right]{\dot{\beta }}_{5}^{2}+\left[\frac{1}{2}m_{6}{\left(L_{HG}\right)}^{2}+\frac{1}{2}I_{6}\right]{\dot{\beta }}_{6}^{2}\\ +\left[\frac{1}{2}m_{7}{\left(\frac{W_{7}}{2}\right)}^{2}+\frac{1}{2}I_{7}\right]{\dot{\beta }}_{7}^{2}+\left[\frac{1}{2}m_{8}{\left(\frac{W_{6}}{2}\right)}^{2}+\frac{1}{2}I_{8}\right]{\dot{\beta }}_{8}^{2}\\ +\left[\frac{1}{2}m_{a}h_{a}^{2}\right]{\dot{\beta }}_{5}^{2}+\left[\frac{1}{2}m_{b}h_{b}^{2}\right]{\dot{\beta }}_{7}^{2} \end{array}$$

In addition, the right circular hinge’s deformation provides elastic energy. The following equations define the elastic energy of the developed structure:

(11) $$E_{V}=\sum_{1}^{n}K_{j}\beta_{j}^{2}=\sum_{1}^{8}K_{j}\beta_{j}^{2}$$

(12) $$\begin{array}{l} E_{V}=\frac{1}{2}(k_{1}+k_{2}+k_{3})\beta_{1}^{2}+\frac{1}{2}k_{4}{\left(\beta_{2}+\beta_{3}\right)}^{2}+\frac{1}{2}k_{5}\beta_{3}^{2}\\ +\frac{1}{2}(k_{6}+k_{7}+k_{8})\beta_{4}^{2}+\frac{1}{2}k_{10}{\left(\beta_{5}+\beta_{6}\right)}^{2}+\frac{1}{2}k_{9}\beta_{6}^{2}\\ \frac{1}{2}k_{11}\beta_{5}^{2}+\frac{1}{2}k_{11}\beta_{7}^{2}+\frac{1}{2}(2k_{12}+2k_{14})\beta_{7}^{2}+\frac{1}{2}4k_{13}\beta_{8}^{2} \end{array}$$

The variables ($\beta_{j}$, $\dot{\beta_{j}}$) used in the equations characterize the rotating angle speed and angle speed of each connection. A sequence of equations can be used to estimate the relationships between rotary angles:

(13) $$\beta_{1}=\frac{d_{in}}{W_{1}}$$

(14) $$\beta_{2}=\frac{d_{2}}{L_{2}}=\frac{(W_{1}+W_{2})\beta_{1}}{L_{2}}$$

(15) $$\beta_{3}=\frac{d_{2}}{L_{2}}=\frac{(W_{1}+W_{2})\beta_{1}}{L_{2}}$$

(16) $$\beta_{4}=\frac{d_{3}}{W_{3}}=\frac{(W_{1}+W_{2})L_{1}\beta_{1}}{W_{3}L_{2}}$$

(17) $$\beta_{5}=\frac{d_{4}}{L_{4}}=\frac{(W_{1}+W_{2})(W_{3}+W_{4})L_{1}\beta_{1}}{W_{3}L_{2}L_{4}}$$

(18) $$\beta_{6}=\frac{d_{4}}{L_{4}}=\frac{(W_{1}+W_{2})(W_{3}+W_{4})L_{1}\beta_{1}}{W_{3}L_{2}L_{4}}$$

(19) $$\beta_{7}=\frac{(W_{1}+W_{2})(W_{3}+W_{4})L_{1}L_{3}\beta_{1}}{W_{1}W_{3}W_{5}L_{2}L_{4}}=\frac{AR.W_{1}}{W_{5}}\beta_{1}$$

(20) $$\beta_{8}=\frac{(W_{1}+W_{2})(W_{3}+W_{4})L_{1}L_{3}\beta_{1}}{W_{1}W_{3}W_{6}L_{2}L_{4}}=\frac{AR.W_{1}}{W_{6}}\beta_{1}$$

The work can be estimated using the input force ${(F}_{in})$ as follows:

(21) $$A=\frac{1}{2}F_{in}d_{in}$$

The link between input displacement and input force can be derived from the equation A = $E_{V}$:

(22) $$\frac{1}{2}F_{in}d_{in}=\frac{1}{2}\left[\begin{array}{l} (k_{1}+k_{2}+k_{3})\frac{1}{W_{1}^{2}}+k_{4}{\left(\frac{\left(W_{1}+W_{2}\right)}{W_{1}}+\frac{\left(W_{1}+W_{2}\right)}{W_{1}}\right)}^{2}\\ +k_{5}{\left(\frac{\left(W_{1}+W_{2}\right)}{W_{1}}\right)}^{2}+(k_{6}+k_{7}+k_{8}){\left(\frac{(W_{1}+W_{2})L_{1}}{W_{1}W_{3}L_{2}}\right)}^{2}\\ +k_{10}{\left(\frac{(W_{1}+W_{2})(W_{3}+W_{4})L_{1}}{W_{1}W_{3}L_{2}L_{4}}+\frac{(W_{1}+W_{2})(W_{3}+W_{4})L_{1}}{W_{1}W_{3}L_{2}L_{4}}\right)}^{2}\\ +k_{9}{\left(\frac{(W_{1}+W_{2})(W_{3}+W_{4})L_{1}}{W_{1}W_{3}L_{2}L_{4}}\right)}^{2}+k_{11}{\left(\frac{(W_{1}+W_{2})(W_{3}+W_{4})L_{1}}{W_{1}W_{3}L_{2}L_{4}}\right)}^{2}\\ +k_{11}{\left(\frac{AR}{W_{5}}\right)}^{2}+(2k_{12}+2k_{14}){\left(\frac{AR}{W_{5}}\right)}^{2}+4k_{13}{\left(\frac{AR}{W_{6}}\right)}^{2} \end{array}\right]d_{in}^{2}$$

To estimate the rigidity of the structure, input rigidity was assigned by the Equation ($K_{in}=F_{in}/d_{in}$), and components were distributed uniformly by $d_{in}^{2}$:

(23) $$\begin{array}{l} K_{in}=(k_{1}+k_{2}+k_{3})\frac{1}{W_{1}^{2}}+k_{4}{\left(\frac{\left(W_{1}+W_{2}\right)}{W_{1}}+\frac{\left(W_{1}+W_{2}\right)}{W_{1}}\right)}^{2}+k_{5}{\left(\frac{\left(W_{1}+W_{2}\right)}{W_{1}}\right)}^{2}\\ +(k_{6}+k_{7}+k_{8}){\left(\frac{(W_{1}+W_{2})L_{1}}{W_{1}W_{3}L_{2}}\right)}^{2}+k_{10}{\left(\frac{(W_{1}+W_{2})(W_{3}+W_{4})L_{1}}{W_{1}W_{3}L_{2}L_{4}}+\frac{(W_{1}+W_{2})(W_{3}+W_{4})L_{1}}{W_{1}W_{3}L_{2}L_{4}}\right)}^{2}\\ +k_{9}{\left(\frac{(W_{1}+W_{2})(W_{3}+W_{4})L_{1}}{W_{1}W_{3}L_{2}L_{4}}\right)}^{2}+k_{11}{\left(\frac{(W_{1}+W_{2})(W_{3}+W_{4})L_{1}}{W_{1}W_{3}L_{2}L_{4}}\right)}^{2}+k_{11}{\left(\frac{AR}{W_{5}}\right)}^{2}\\ +(2k_{12}+2k_{14}){\left(\frac{AR}{W_{5}}\right)}^{2}+4k_{13}{\left(\frac{AR}{W_{6}}\right)}^{2} \end{array}$$

The mechanism can store kinetic and elastic energies ($E_{K}$, and $E_{V}$). The Lagrangian Equation $L=E_{K}-E_{V}$ is derived by replacing the kinetic and potential energy:

(24) $$L=E_{K}-E_{V}$$

(25) $$\sum_{1}^{8}\left\{\frac{d}{dt}\left(\frac{\partial L}{\partial \overset{\cdot }{\beta_{j}}}\right)-\frac{\partial L}{\partial \beta_{j}}=Q_{j}\right\}$$

The following equation defines the equation of motion:

(26) $$\bar{M}\overset{\cdot \cdot }{\beta_{1}}+\bar{K}\beta_{1}=0$$

in which:

(27) $$\begin{array}{l} \bar{M}=m_{1}\cdot {\left(\frac{W_{1}+W_{2}}{2}\right)}^{2}+I_{1}+m_{2}{\left(\frac{L_{BC}\left(W_{1}+W_{2}\right)}{L_{2}}\right)}^{2}+I_{2}{\left[\frac{\left(W_{1}+W_{2}\right)}{L_{2}}\right]}^{2}\\ +m_{3}{\left(\frac{L_{CD}\left(W_{1}+W_{2}\right)}{L_{2}}\right)}^{2}+I_{3}{\left[\frac{\left(W_{1}+W_{2}\right)}{L_{2}}\right]}^{2}+m_{4}\cdot {\left(\frac{\left(W_{1}+W_{2}\right)\left(W_{3}+W_{4}\right).L_{1}}{2W_{3}.L_{2}}\right)}^{2}\\ +I_{4}{\left(\frac{\left(W_{1}+W_{2}\right).L_{1}}{W_{3}.L_{2}}\right)}^{2}+m_{5}{\left(\frac{L_{FG}\left(W_{1}+W_{2}\right)\left(W_{3}+W_{4}\right).L_{1}}{W_{3}L_{2}L_{4}}\right)}^{2}\\ +I_{5}{\left(\frac{\left(W_{1}+W_{2}\right)\left(W_{3}+W_{4}\right).L_{1}}{W_{3}L_{2}L_{4}}\right)}^{2}+m_{6}{\left(\frac{L_{HG}\left(W_{1}+W_{2}\right)\left(W_{3}+W_{4}\right).L_{1}}{W_{3}L_{2}L_{4}}\right)}^{2}\\ +I_{6}{\left(\frac{\left(W_{1}+W_{2}\right)\left(W_{3}+W_{4}\right).L_{1}}{W_{3}L_{2}L_{4}}\right)}^{2}+m_{7}{\left(\frac{AR\cdot W_{1}}{2}\right)}^{2}+I_{7}{\left(\frac{AR\cdot W_{1}}{W_{5}}\right)}^{2}\\ +2m_{8}{\left(\frac{AR\cdot W_{1}}{2}\right)}^{2}+I_{8}{\left(\frac{AR\cdot W_{1}}{W_{6}}\right)}^{2}+m_{a}{\left(\frac{h_{a}\left(W_{1}+W_{2}\right)\left(W_{3}+W_{4}\right).L_{1}}{W_{3}L_{2}L_{4}}\right)}^{2}\\ +m_{b}{\left(\frac{h_{b}AR\cdot W_{1}}{W_{5}}\right)}^{2} \end{array}$$

(28) $$\begin{array}{l} \bar{K}=\left(k_{1}+k_{2}+k_{3}\right)+4k_{4}{\left(\frac{W_{1}+W_{2}}{L_{2}}\right)}^{2}+k_{5}{\left(\frac{W_{1}+W_{2}}{L_{2}}\right)}^{2}\\ +\left(k_{6}+k_{7}+k_{8}\right){\left(\frac{\left(W_{1}+W_{2}\right)L_{1}}{W_{3}L_{2}}\right)}^{2}+4k_{10}{\left(\frac{\left(W_{1}+W_{2}\right)\left(W_{3}+W_{4}\right)L_{1}}{W_{3}L_{2}L_{4}}\right)}^{2}\\ +k_{9}{\left(\frac{\left(W_{1}+W_{2}\right)\left(W_{3}+W_{4}\right)L_{1}}{W_{3}L_{2}L_{4}}\right)}^{2}+k_{11}{\left(\frac{\left(W_{1}+W_{2}\right)\left(W_{3}+W_{4}\right)L_{1}}{W_{3}L_{2}L_{4}}\right)}^{2}\\ +k_{11}{\left(\frac{AR.W_{1}}{W_{5}}\right)}^{2}+2k_{12}{\left(\frac{AR.W_{1}}{W_{5}}\right)}^{2}+2k_{14}{\left(\frac{AR.W_{1}}{W_{5}}\right)}^{2}+4k_{13}{\left(\frac{AR.W_{1}}{W_{6}}\right)}^{2} \end{array}$$

The resonant frequency of the developed positioner is determined using the abovementioned equation.

(29) $$f=\frac{1}{2\pi }\sqrt{\frac{\bar{K}}{\bar{M}}}$$

### 4.2. Working Stroke Analysis

The working travel of the proposed positioner can be determined by multiplying AR and AR. It is vital to note that this estimate ignores the effects of axial stress and shearing, focusing solely on bending tension. As a result, the equation for calculating the stress is expressed as:

(30) $$Max(\sigma )\leq \frac{\sigma_{t}}{S_{e}}$$

$\sigma_{t}$ and $S_{e}$ are the tensile yield strength and safety factor. In addition, to ensure the strength of the material, $S_{e}$ is proposed to select 1.5. Furthermore, the highest stress occurs when the highest angle deformation achieves its maximum worth. Thus, the highest stress is calculated using the following equation:

(31) $$\sigma_{\max}=\frac{4Er^{2}K_{c}}{f(\xi )t^{2}}\varphi_{\max}$$

In addition, the non-dimensional focus element and $K_{c}$ denote the compliance element:

(32) $$K_{c}={\left(1+\xi \right)}^{9/20}$$

(33) $$\xi =\frac{t}{2r}$$

(34) $$f(\xi )=\frac{1}{2\xi +\xi^{2}}\left(\frac{3+4\xi +2\xi^{2}}{\left(1+\xi )(2\xi +\xi^{2}\right)}+\frac{6(1+\xi )}{{\left(\sqrt{2\xi +\xi^{2}}\right)}^{3}}\tan^{-1}\sqrt{\frac{2+\xi }{\xi }}\right)$$

The most significant input displacement is denoted as follows, and the maximum value represents the output deformation of the mobility platform. The computation for defining the highest displacement angle involves the following:

(35) $$\theta_{\max}=\frac{AR\times d_{in}^{\max}}{2\times W}$$

Calculating the maximal input displacement is accomplished by combining Equations (31) and (32) while simultaneously considering Equations (34) and (35):

(36) $$\frac{\sigma_{t}}{S_{e}}\geq \frac{4Er^{2}K_{c}}{f(\xi )t^{2}}(\frac{AR\times d_{in}^{\max}}{2\times W})$$

(37) $$d_{in}^{\max}\leq \frac{\sigma_{y}f(\xi )t^{2}2W}{4S_{e}Er^{2}K_{c}AR}$$

To determine the maximum displacement, the parameters of the proposed material specified in Table 1 are substituted into Equation (37):

(38) $$d_{in}^{\max}\leq 36.5\;µm$$

Considering the mechanism’s amplification ratio, the stage’s output displacement can be determined to be 613.2 µm. Due to the mechanism’s symmetry, the platform’s reachable workspace can be expressed as 613.2 µm × 613.2 µm.

### 4.3. Analytical Modelling Validation

**Figure 8:**
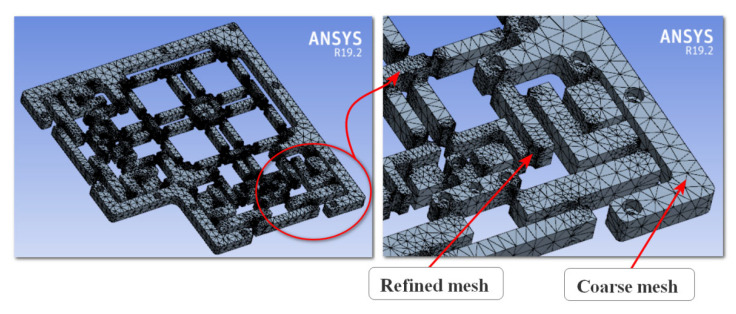
Mesh model for the suggested mechanism.

**Figure 9:**
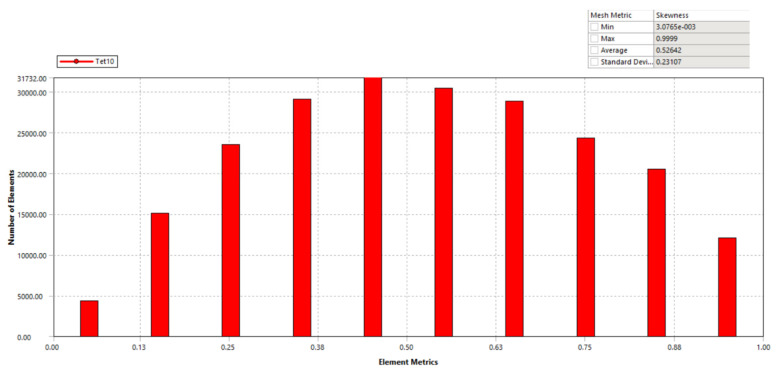
Skewness-based meshing quality of the developed mechanism.

**Figure 10:**
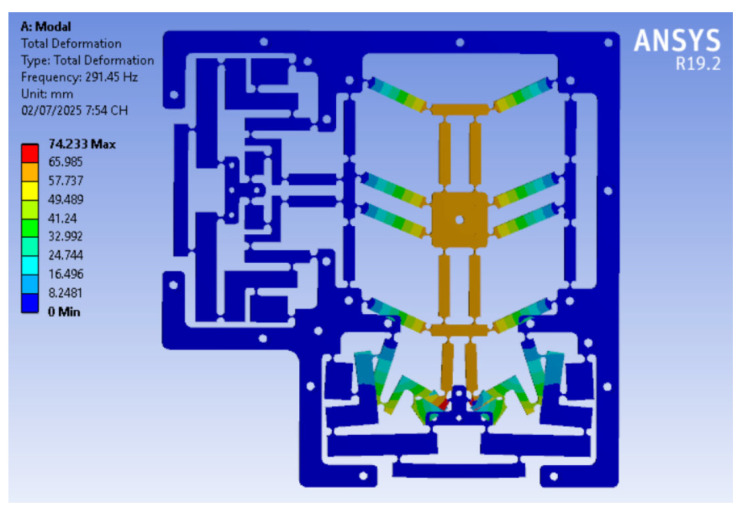
The FEA-based natural frequency.

The positioner design model was initially constructed using a coarse mesh to validate the analytical outcome. To enrich the computation accuracy, the mesh model was treated at the designated locations of the circular joints, as illustrated in Figure 8. The simulation mesh conditions for the developed positioner included 219,462 mesh elements and 371,608 mesh nodes. Additionally, the fixed support’s boundary condition was at the flexure positioner’s holes, and no input force or displacement was provided for calculating the first natural frequency. The Skewness standard [40] was subsequently utilized to assess the meshing attribute. According to this standard, the resultant value of a modest worth was 0.52642, as illustrated in Figure 9. This number guaranteed the attainment of a suitable mesh for the simulated process of the proposed positioner. The FEA resonant frequency yielded 291.45 Hz, as illustrated in Figure 10. The achieved analytical resonant frequency was 298.55 Hz. In addition, the analytical resonance frequency was confirmed using FEA analysis.

Table 2 shows a 2.436% theoretical-FEM imprecision. Therefore, the proposed modelling approach can assess the positioner output attribute.

### 4.4. Parameter Optimisation of the XY Positioner

The new structure of the 02-DOF mechanism was created for non-resonant vibration-aided fine CNC milling. The initial phase involved developing an analytic model for the suggested positioner to evaluate the output feature through the PRBM and Lagrangian methods. The VAM stage was arranged in parallel, allowing for the independent engagement of each direction (X and Y). Analyzing a one-directional approach suffices for the overall design due to its symmetric construction. The initial resonant frequency modes were either constrained or augmented to decrease the resonance circumstance amongst the PEA actuator and the proposed mechanism. Selecting the initial resonant frequency with the highest possible magnitude is recommended to enhance the responsiveness of the positioners. Therefore, optimizing the resonant frequency is advisable to improve the response speed, alleviate resonance phenomena in the positioner, and boost the stiffness of the recommended structure in non-resonant vibration-aided fine milling. The significant aim of this study is to improve the natural frequency, which is succinctly identified as follows:

Find the design parameter: *X* = [*G*, *K*, *N*, *W*, *Q*, *Y*, *P*]

(39) Maximize f(X)

Constraint:

(40) f(X)>290 Hz

Limits of key factors (unit: mm):

(41) $$\left\{\begin{array}{l} 1\leq G\leq 1.2\\ 0.9\leq K\leq 1\\ 0.85\leq N\leq 0.9\\ 0.8\leq E\leq 0.85\\ 0.7\leq Q\leq 0.8\\ 20\leq Y\leq 28\\ 15\leq P\leq 19 \end{array}\right.$$

where *f (X)* represents the natural frequency, while the key parameters *G*, *K*, and *N*, as well as *E*, signify the thickness of right circular hinges on floors 1 through 4 of the hybrid displacement amplifier, respectively, as illustrated in various colors in the initial design diagram in Figure 4. Furthermore, *Q* represents the proposed joint thickness on the parallel leading structure. To ensure efficient displacement transmission energy, the thicknesses of the right circular hinges varied across different floors. *Y* and *P* present the distances between two right circular hinges on the first floor and the third floor, respectively.

**Figure 11:**
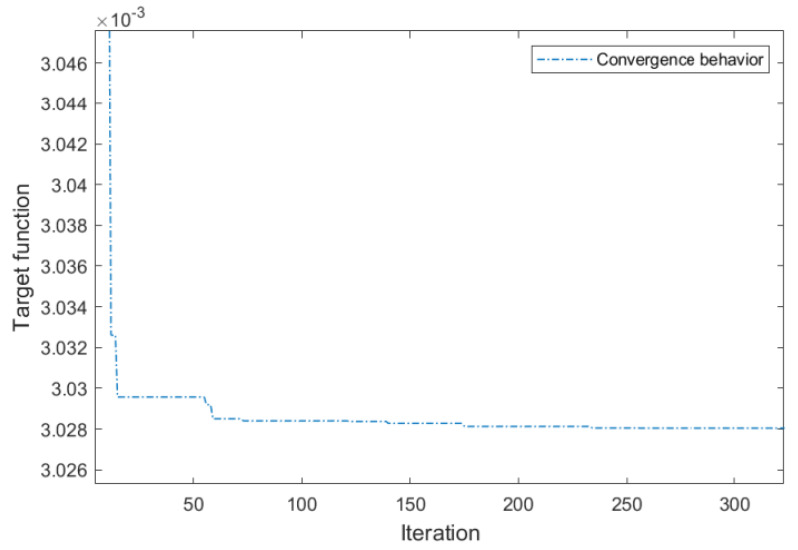
Convergence plot of the proposed algorithm.

Based on the hybrid analytical approach of the PRBM and the Lagrange technique for building the chain of analytical Equations (1)–(29), MATLAB R2021b was utilized to optimize the main factors of the suggested positioner using the Firefly algorithm. Specifically, the Firefly algorithm was chosen for its intelligent behaviors and efficacy in addressing highly nonlinear and multimodal optimization challenges, particularly in mechanical design. The algorithm settings were established as follows: population size (n) of 20, maximum iterations of 1000, a randomization parameter (α) of 0.5, and a light absorption coefficient (γ) of 1. In addition, the convergence criterion was established as the absolute change in the objective function value being less than 10^−6^. The convergence plot of the offered algorithm is portrayed in Figure 11.

**Figure 12:**
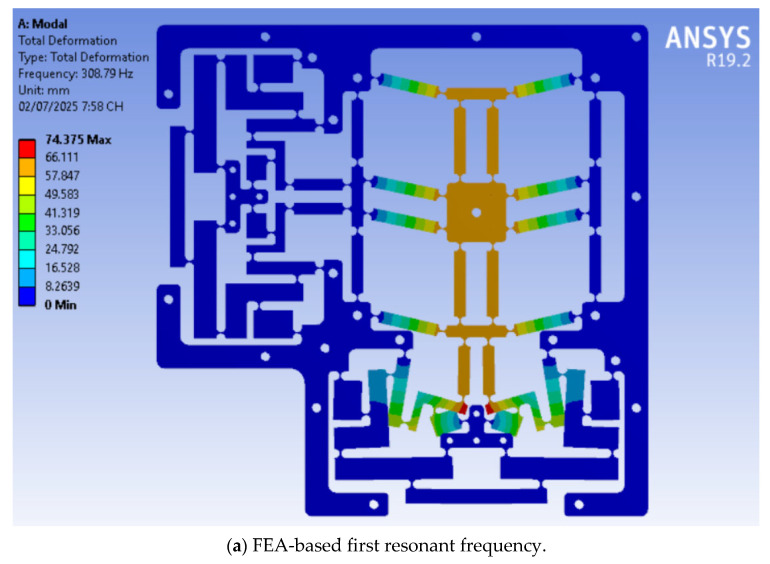

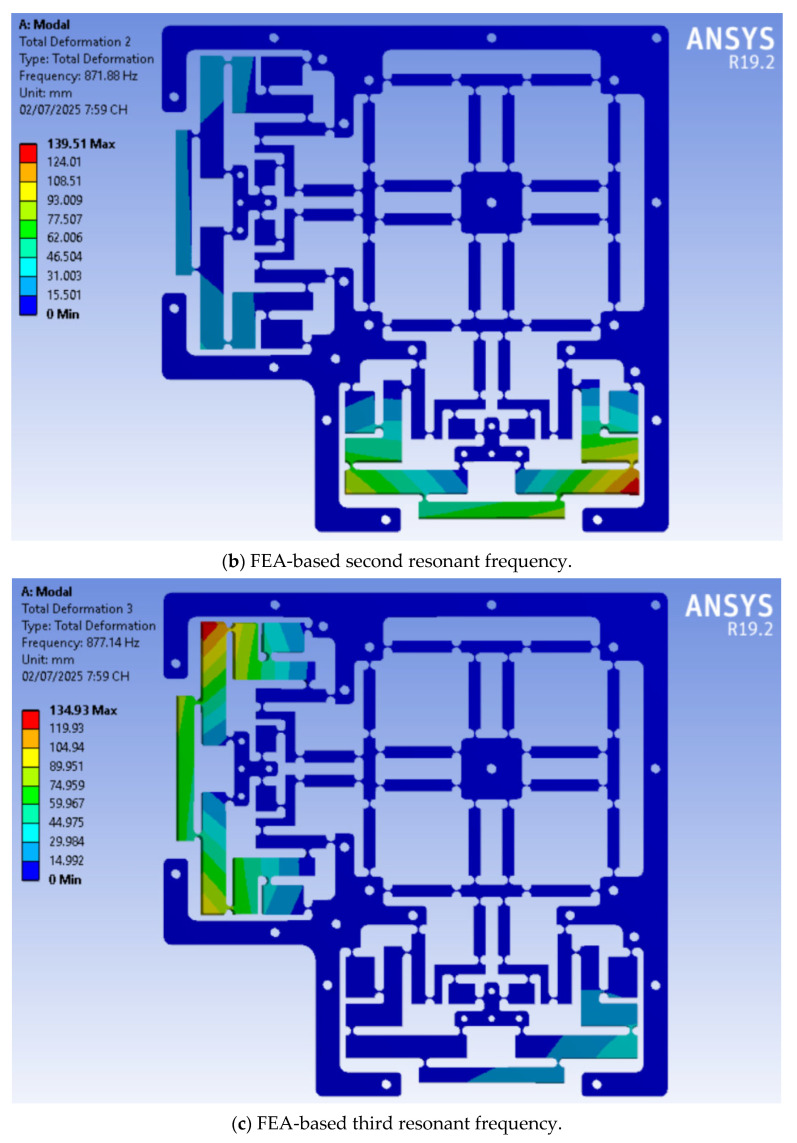

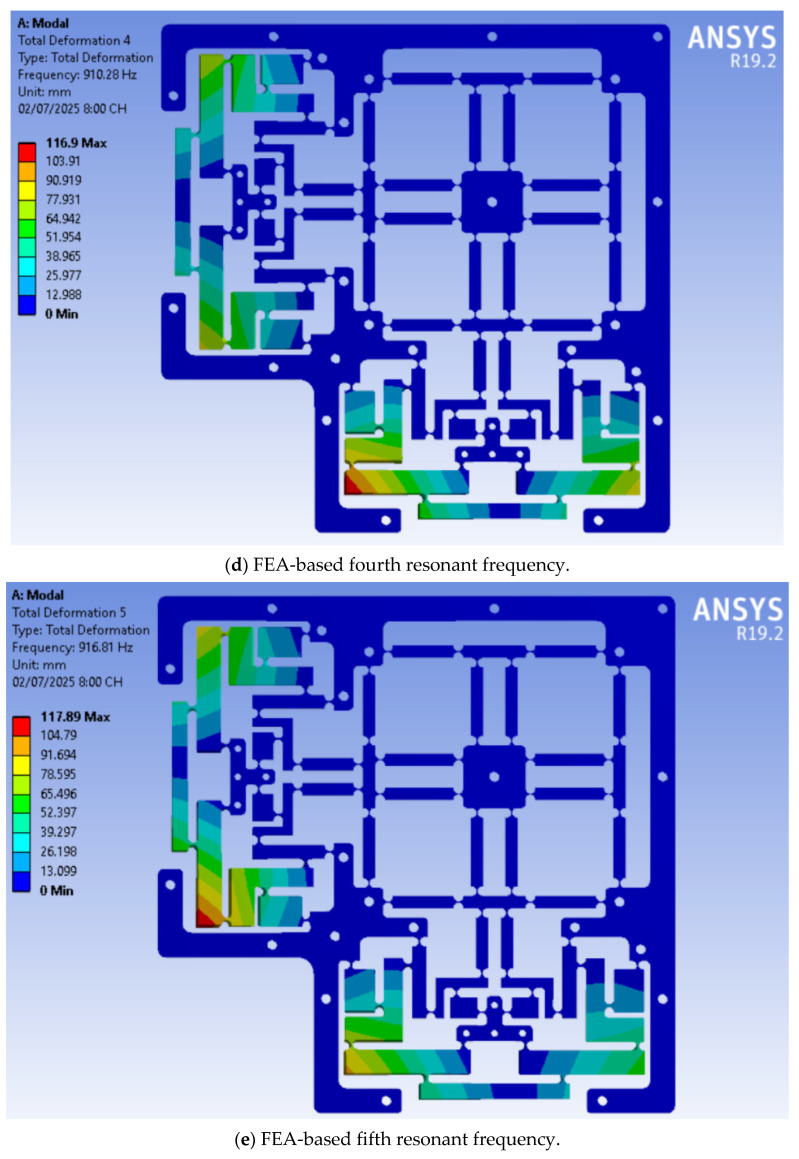

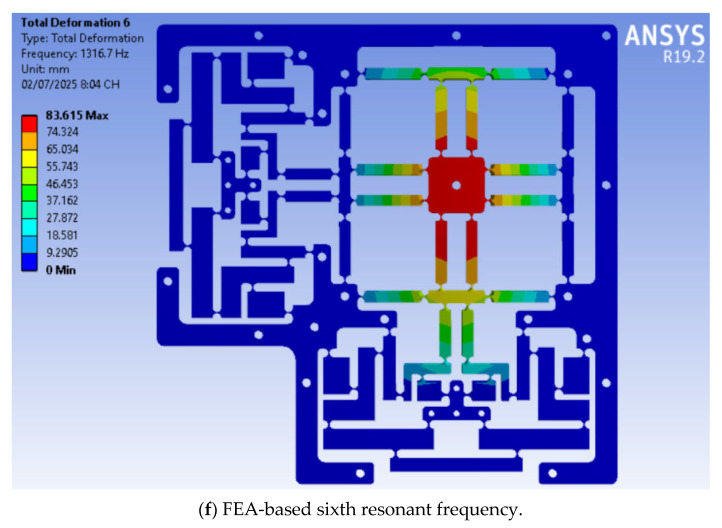
Optimized FEA resonant frequencies at the first six modes.

The optimal parameters for the developed positioner are determined at: *G* = 1.2 mm; *K* = 1 mm; *N* = 0.9 mm; *E* = 0.85 mm; *Q* = 0.8 mm; *Y* = 23.63 mm; and *P* = 15 mm. The results suggest that the optimized resonant frequency is approximately 318.16 Hz. The optimal result was confirmed through FEA analysis. The ideal parameters were used to develop a 3D model to validate the optimized consequence. The results reveal that the initial resonance frequency, defined through the FEA study, is 308.79 Hz, as shown in Figure 12a. The difference between the optimized results and those obtained from the FEA is 3.04%, as shown in Table 3. Additionally, the FEA frequency values of the positioner for the initial six modes are recorded at 308.79 Hz, 871.88 Hz, 877.14 Hz, 910.28 Hz, 916.81 Hz, and 1316.7 Hz, as portrayed in Figure 12a–f.

### 4.5. Experimental Results

#### 4.5.1. Resonant Frequency Testing

**Figure 13:**
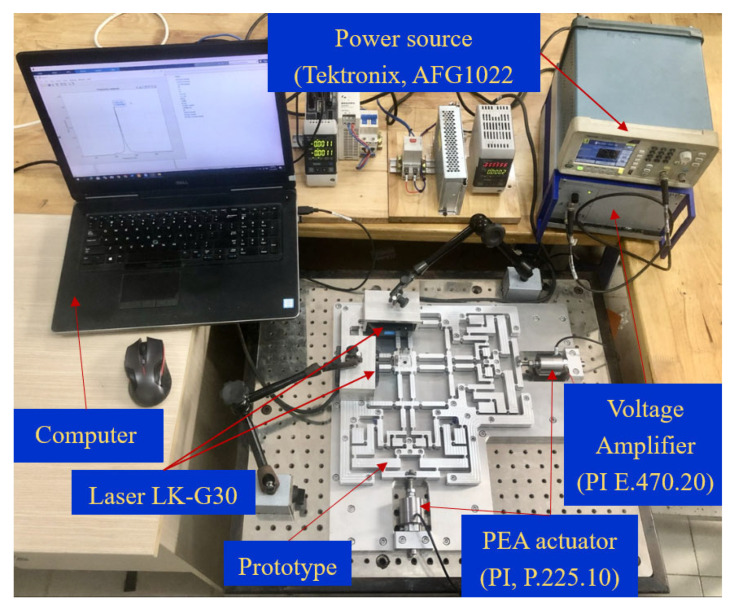
Installation of an experiment to ascertain the resonant frequency.

**Figure 14:**
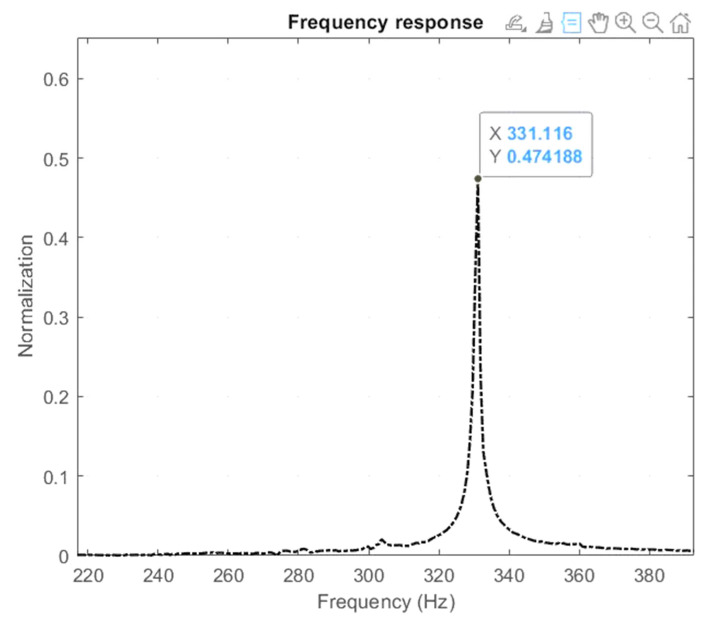
Results of the experimental resonant frequency.

The arrangement of the experiment, illustrated in Figure 13, was designed to test the theoretical and FEA computations of the mechanism’s output properties. Keyence’s LK-G30 laser displacement sensor accurately recorded the vibrations of the center platform in the X–Y axis to within 0.05 µm. The hammer testing methodology defined the mechanism’s primary resonant frequency, which involved analyzing data captured by the LK-G30 laser sensor, Keyence company, Japan. The laser displacement sensor operated under software control. The obtained information was analyzed using the quick Fourier transform procedure in MATLAB software R2021b. Figure 14 depicts the experimental results, with the initial peak of the frequency spectrum found at 331.116 Hz. The result indicated a 7.23% difference between the FEA and experiment results, as shown in Table 4. The discrepancy was modest and narrowly matched the FEA result.

Table 5 shows that the resonant frequency of the ideal design improved by 5.95% compared to the first design calculated using FEA analysis.

#### 4.5.2. Examination of Experimental CNC Machining on a Fabricated Optimization Positioner

**Figure 15:**
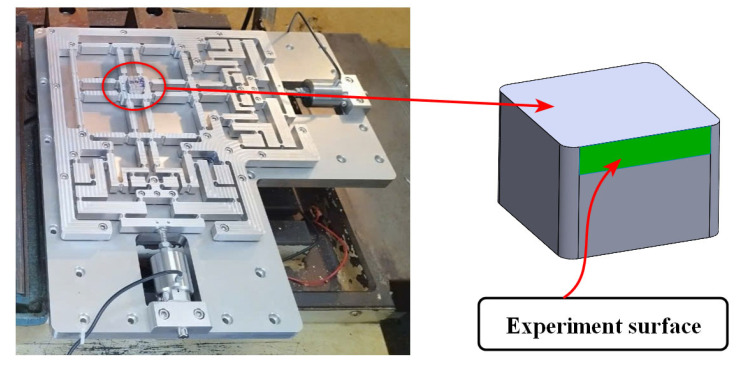
The manufactured surface for the experimental material sample.

Figure 15 depicts the experimental configuration established for conducting side-face CNC milling tests. This arrangement aims to evaluate the characteristics of the suggested integration fabricating method compared to the traditional CNC milling process. The experimental fabrication process employs fine CNC milling machinery, namely, the VMC-650 (Huey Long company, Taichung, Taiwan). The material sample is a rigid SKD11 material, fabricated for a plastic mould. The vibration milling equipment with assisted motion operates in two orientations: separately or simultaneously. The instructions follow the feeding specifications based on the X or Y pathways. This experiment investigates the impact of vibration-aided frequency on the feed path utilizing a PEA motor. The aim of the experimental process is to assess the surface roughness of the material samples fabricated using the traditional fabricating CNC method, compared to those created using assisted vibration manufacturing techniques.

**Figure 16:**
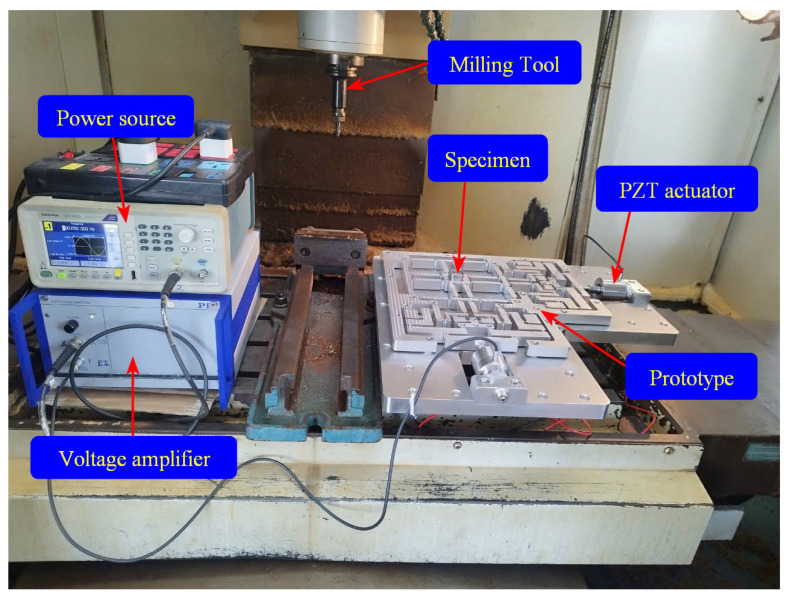
Vibration-aided fine CNC milling experimental layout for the SKD 11 specimen.

The experiment utilized fixed fabricating parameters: a dry cutting condition, an endmill tool diameter of 8 mm, a manufacturing thickness of 8 mm, a spindle velocity of 2000 rpm, and a feed rate of 80 mm/min. Additionally, the fabricated prototype of the developed positioner was mounted on the intermediate plate, which, in turn, was secured to the machine table of the CNC machine. The specimen was fixed to the prototype on the central platform using a screw. PEA actuators with a maximum displacement of 15 µm were employed to assess vibration frequencies ranging from 100 to 1000 Hz. The maximum voltage applied was 10 V. A frequency generator (AFG1022, Tektronix, Beaverton, OR, USA) delivered the investigated frequency for the PEA motor, the P-225.10 (PI, Karlsruhe, Germany), enhancing the CNC milling vibration. This experimental process evaluated the output feature of the manufactured specimens, as illustrated in Figure 16.

**Figure 17:**
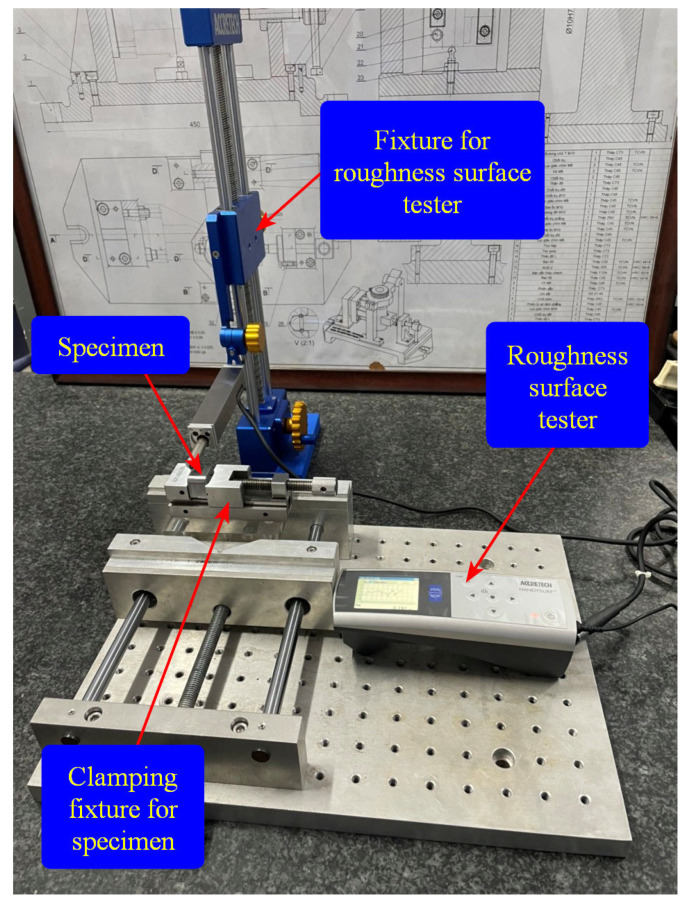
Experiment fixture for side-face roughness testing of manufactured specimens.

**Figure 18:**
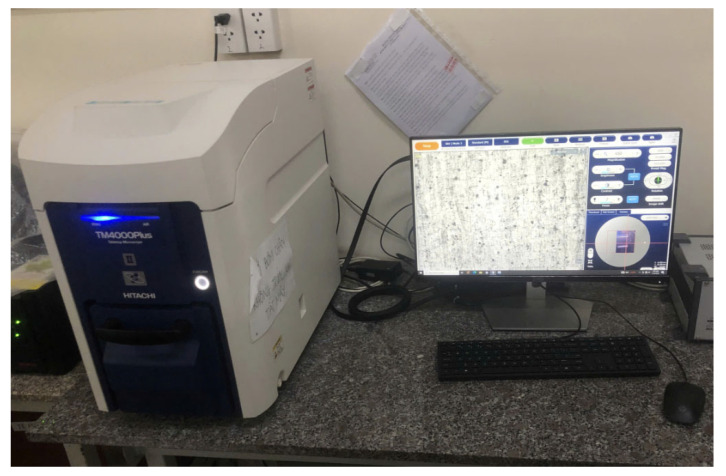
The experimental layout used to check the surface morphology of the manufactured surfaces.

**Figure 19:**
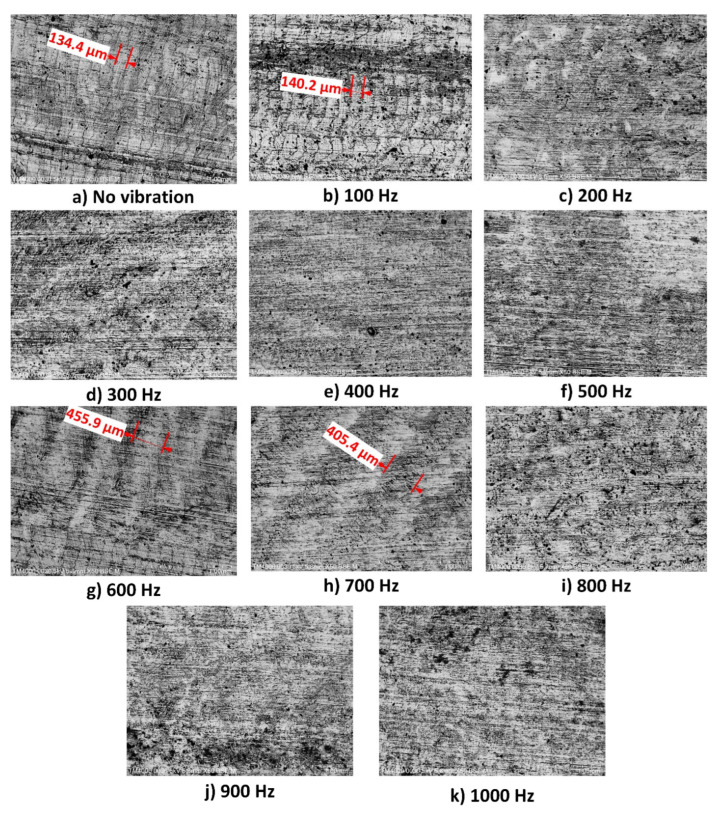
Surface morphology in the investigated frequency range [100 Hz, 1000 Hz].

Figure 17 shows the experimental arrangement used to evaluate the surface roughness of the fabricated sample’s side faces. A handysurf^+^ roughness testing device (Accretech, Tokyo Seimitsu, Hachiōji-shi, Tokyo, Japan) was employed to measure the surface roughness of the sample within the frequency range of 100 Hz to 1000 Hz. The experimental consequences for assessing the face roughness of the milled specimen are presented in Table 6. The surface morphology of the non-vibration-assisted milled surface of the milled sample was captured using SEM, namely, a Hitachi TM4000Plus, Hitachi High-Tech Corporation, Tokyo, Japan, as depicted in Figure 18. In addition, the surface morphologies of vibration-assisted milled surfaces in non-vibration-assisted milled surface and in a frequency range [100 Hz, 1000 Hz] are shown in Figure 19a–k. The attained optimal surface roughness (Ra) was 0.187 µm at a frequency of 400 Hz. In some cases, there are some step marks or terraces on the machined surface. With “no vibration” and 100 Hz cases, as shown in Figure 19a,b, the average step widths are 134.4 μm and 140.2 μm, which are quite close to each other. This is why the surface roughness changes only slightly, from 0.638 μm to 0.601 μm. Interestingly, from 200 Hz to 1000 Hz, the step mark disappears or becomes blurred with a greater step width, leading to a sudden reduction in surface roughness. This phenomenon indicates the advantage of applying vibration during the milling process. Specifically, at 400 Hz, as shown in Figure 19e, there is no step mark. The surface also presents a smooth appearance compared to other cases. This is the reason for the lowest surface roughness of 0.187 μm, which is the optimal result. Consequently, the non-resonant vibration-assisted CNC fine milling technique improved the face roughness of the sample within an optimal frequency breadth. In addition, the investigated frequency based on the developed positioner for the SKD material was a good reference for fabricating engineers to enhance the quality of the surfaces of fine-manufactured components in the mould manufacturing field.

As for previous studies of the surface roughness efficiency of non-resonant vibration-assisted specimens, Pham et al. [20] designed the 02-DOF positioner for non-resonant vibration-assisted milling using an AL6061 material sample. The milled specimen’s ideal surface roughness was 0.638 µm at 325 Hz. Dang et al. [21] proposed a novel 02-DOF stage integrated with a symmetric four-lever displacement amplifier, which was utilized for non-resonant vibration-assisted milling for the 2083 specimen. The examined frequencies ranged from 700 to 1000 Hz, and the optimal experiment frequency of the developed mechanism was 900 Hz with 0.321 µm. This study provided a novel design for a compliant 02-DOF stage, utilizing an SKD11 material specimen with a Ra of 0.187 µm at an investigation frequency of 400 Hz, with frequencies tested within the 100 Hz to 1000 Hz range. In addition, the first natural frequency, working stroke, and amplification of the present study were compared with those of previous studies, as demonstrated in Table 7. 

## 5. Conclusions

This study develops a new structure for a flexure XY positioner used in non-resonant vibration-aided CNC milling. The XY positioner was initially constructed with a unique hybrid displacement amplifier that combines a double symmetric amplifier with one-lever and Scott–Russell mechanisms in a perpendicular series configuration. A rapid PRBM integration method was employed alongside the Lagrangian methodology to analyze the quality response of the proposed platform. The analytical and FEA resonant frequencies were found to be 298.55 and 291.45 Hz, respectively. The accuracy of the analytical form was assessed via FEA analysis, which revealed a 2.436% discrepancy. As a result, the hybrid analytical method proved reliable in quickly analyzing the quality response of the created platform.

Third, the Firefly algorithm was employed to optimize the significant design variables of the developed stage, thereby enhancing the quality response to conform with the established chain of equations. The optimal values for the crucial parameters were *G* = 1.2 mm, *K* = 1 mm, *N* = 0.9 mm, *E* = 0.85 mm, *Q* = 0.8 mm, *Y* = 23.63 mm, and *P* = 15 mm. The analytically determined optimal resonant frequency was 318.16 Hz. Moreover, the optimal consequence was validated using FEA analysis. The vital components were utilized to create a 3D model to confirm the optimized analytical consequence using the FEM approach. The FEA optimum value was 308.79 Hz, with a 3.04% difference between the analytical and FEA estimates. The experimental technique was employed to validate the optimal results obtained from the FEA analysis. The results indicated that the experimental frequency was 331.116 Hz, representing a 7.23% deviation from the ideal value calculated using FEA analysis.

A machining experiment demonstrated that vibration-assisted milling enhances surface roughness standards compared to traditional milling. The testing method used vibration frequencies varying from 100 to 1000 Hz. At 400 Hz, and the specimen achieved an optimal surface roughness of 0.187 µm. These findings support non-resonant vibration-assisted fine CNC machining for improved surface quality. Compared to previous studies, the developed novel structure exhibits good characteristics, including minor surface roughness and a high working stroke, with a suitable natural frequency.

## Figures and Tables

**Table 1:** Key elements of the suggested positioner (unit: mm).

Sign	Worth	Unit	Sign	Worth	Unit
*G*	1 ≤ *G* ≤ 1.2	mm	m	8	mm
*K*	0.9 ≤ *K* ≤ 1	mm	n	15	mm
*N*	0.85 ≤ *N* ≤ 0.9	mm	o	9	mm
*E*	0.8 ≤ *E* ≤ 0.85	mm	a	330.5	mm
*Q*	0.7 ≤ *Q* ≤ 0.8	mm	b	330.5	mm
*Y*	20 ≤ *Y* ≤ 28	mm	c	231	mm
*P*	15 ≤ *P* ≤ 19	mm	d	231	mm
j	40	mm	g	16	mm
k	40	mm	h	142	mm
l	8	mm	i	40	mm

**Table 2:** Validation of the theoretical al result against the FEA result.

Feature	Theoretical	FEA	Imprecision (%)
*f* (Hz)	298.55	291.45	2.436%

**Table 3:** Association of the optimized outcome based on FEA results.

Feature	Analytical	FEA	Error (%)
*f* (Hz)	318.16	308.79	3.04%

**Table 4:** Verification of experimental and finite element analysis results.

Attribute	FEA	Experiment	Inaccuracy (%)
*f* (Hz)	308.79	331.116	7.23%

**Table 5:** Comparison of the initial natural frequency enhancement between the optimal and baseline designs.

Attribute	Baseline Design	Optimized Design	Enhancement (%)
*f* (Hz)	291.45	308.79	5.95%

**Table 6:** Achieved surface roughness between the non-resonant VAM method and traditional CNC milling.

No.	Investigated Frequency (Hz)	Surface Roughness (µm) (VAM)	Surface Roughness (µm) (Traditional Milling)
1	100	0.601	0.638
2	200	1.067	0.638
3	300	0.196	0.638
4	400	0.187	0.638
5	500	0.3	0.638
6	600	0.308	0.638
7	700	0.283	0.638
8	800	0.475	0.638
9	900	1.855	0.638
10	1000	0.337	0.638

**Table 7:** Comparison of the present structure and different prior studies.

Studies	Total Dimensions	First Resonant Frequency (Hz)	Workspace	Amplification Ratio
[25]	N/A	168.22	150.3 µm × 147.9 µm	7.48
[41]	451 mm × 451 mm	112.1	787.63 μm × 794.5 μm	7.79
[24]	N/A	220.43	112 µm × 89 µm	7.55
This study	330.5 mm × 330.5 mm	308.79	613.2 µm × 613.2 µm	16.8

## Data Availability

The original contributions presented in the study are included in the article; further inquiries can be directed to the corresponding author.
